# The prognostic role of tumor mutation burden on survival of breast cancer: a systematic review and meta-analysis

**DOI:** 10.1186/s12885-022-10284-1

**Published:** 2022-11-17

**Authors:** Liyuan Ke, Su Li, Hongxia Cui

**Affiliations:** Cancer Hospital of China Medical University, Liaoning Cancer Hospital & Institute, Shenyang, China

**Keywords:** Tumor mutation burden, Overall survival, Progression-free survival, Breast cancer, Meta-analysis

## Abstract

**Background:**

As a potential genetic biomarker, tumor mutation burden (TMB) has made progress in numerous tumors. There are limited data regarding TMB and its prognostic role is controversial in breast cancer. This systematic review and meta-analysis were conducted to assess the prognostic value of TMB on survival of breast cancer.

**Methods:**

The databases PubMed, Embase, Web of Science, and Cochrane Library were searched for articles published through May 31, 2022. Moreover, effective data were extracted from included studies and calculated pooled effects of hazard ratio (HR) for overall survival (OS) and progression-free survival (PFS) by STATA 16.0. Heterogeneity was conducted by the *I*^*2*^ statistic and *p-*value. Using publication bias evaluation, sensitivity analysis, and subgroup analysis, the origin of heterogeneity was further investigated.

**Results:**

They were up to 1,722 patients collected from sixteen cohorts for this analysis. The pooled effects of HR for both OS (HR: 1.14, 95% CI: 0.83,1.58, *p* > 0.01) and PFS (HR: 0.96, 95% CI: 0.53,1.71, *p* > 0.01) indicated no statistically significant difference in the high TMB and low TMB group. In immune checkpoint inhibitors (ICIs) subgroup, high TMB patients demonstrated benefit of OS (HR: 0.72, 95% CI: 0.59,0.87, *p* = 0.001) and PFS (HR: 0.52, 95% CI: 0.35,0.77, *p* < 0.001), whereas difference was not statistically significant in the non-ICIs subgroup (OS, HR:1.76, 95% CI: 0.97,3.20, *p* = 0.062; PFS, HR:2.31, 95% CI: 0.89,5.97, *p* = 0.086). In addition, sensitivity analysis revealed that the pooled effects were stable. The funnel plot and Begg's test suggested the absence of publication bias.

**Conclusion:**

Meta-analysis revealed that the prognostic relevance of TMB in breast cancer is limited in scope. High TMB may be associated with longer survival only in ICIs-based treatment, but the association is not evident in non-ICIs-based treatment.

**Trial registration:**

[https://www.crd.york.ac.uk/PROSPERO], Prospective Register of Systematic Reviews (PROSPERO), identifier: CRD42022342488.

**Supplementary Information:**

The online version contains supplementary material available at 10.1186/s12885-022-10284-1.

## Introduction

In accordance with global cancer statistics, nearly 2.3 million new cases of breast cancer have been diagnosed in 2020 [1]. In addition, breast cancer has relatively higher treatment response rates and longer 5-year survival rates than other tumors [2]. Chemotherapy, endocrine therapy, and targeted therapy play a crucial role in the treatment of breast cancer, and the choice of appropriate treatment strategies depends on the expression level of molecular markers for estrogen receptors (ER) or progesterone receptors (PR) and human epidermal growth factor 2 (HER2) [3]. With the most recent developments in complex genomics, the prognostic and predictive biomarkers in breast cancer are not limited to the aforementioned protein expression, some genetic-based biomarkers are currently being developed [4]. Prospective studies of the biomarkers PIK3CA and germline BRCA1/2 alterations in breast cancer drive the approval of the targeted drugs PI3K inhibitor and PARP inhibitor [5, 6]. Increasing the number of predictive and prognostic biomarkers will improve the quality of oncology care. As an extremely promising genetic biomarker, tumor mutational burden (TMB) is a complement to conventional biomarkers for identifying additional patients who may benefit from treatment options.

TMB measures the number of somatic mutations within a tumor genome by the unit of mutations per megabase (Mut/Mb). The high TMB is associated with a high neoantigen burden, which makes tumors much more immunogenic [7]. In comparison to immunogenic tumors, the TMB value of breast cancer is intermediate, with a median mutation rate of 2.63 Mut/Mb [8, 9]. There is higher TMB in triple-negative breast cancer (TNBC) than in ER ( +) or HER2 ( +) cancers [10, 11]. The predictive role of TMB in TNBC was exhibited in the IMpassion130 phase III clinical trial and exploratory analysis. Emens et al. confirmed that a higher TMB was associated with an improvement in overall survival in the atlizumab plus albumin-paclitaxel group [12]. In a clinical study on patients with HER2 ( +) metastatic breast cancer, the results revealed a statistically significant difference between the median overall survival of those in the low and high TMB groups (44.9 months vs. 85.8 months) [13]. TMB has achieved certain processes in predicting response to immune checkpoint inhibitors [14]. Following phase II results from KEYNOTE-158, the Food and Drug Administration approved pembrolizumab for the treatment of patients with high tumor mutational burden solid tumors [15]. And this study covered a variety of tumors but not breast cancer. Furthermore, the KEYNOTE-119 trial recruited metastatic TNBC patients to explore the association of TMB and clinical outcomes [16]. The research team noted a potential positive association between TMB and clinical benefit with pembrolizumab in patients with metastatic TNBC. Similarly, a retrospective analysis of 62 patients with metastatic breast cancer revealed that high TMB patients benefited from ICIs therapy [17]. As illustrated by the results from the phase II TAPUR trial, pembrolizumab monotherapy demonstrated antitumor activity in patients with high TMB metastatic breast cancer [18]. TMB has been analyzed to predict the clinical outcome of breast cancer patients treated with neoadjuvant chemotherapy, targeted therapy, or standard chemotherapy, in addition to immunotherapy. In terms of neoadjuvant therapy, there are two opposing views. One view held that TMB levels are higher in the non-pathological complete response group, while the opposing view held that TMB levels are higher in the pathological complete response group [19, 20]. In addition to neoadjuvant therapy, the association between TMB and the efficacy of targeted therapy, such as anti-HER2 therapy, has been analyzed. Chen's study revealed a statistically significant inverse association between TMB and PFS, and TMB could be used to evaluate the efficacy of pyrotinib in HER-positive metastatic breast cancer [21]. Park suggested that high TMB could provide an outstanding prediction of standard chemotherapy [13]. In contrast, Barroso-Sousa and Samstein argued that higher TMB was not associated with better outcomes and was even associated with a poorer response to non-ICIs treatments [8, 22].

In breast cancer, there is limited data regarding TMB and its prognostic role is controversial. Consequently, this systematic review and meta-analysis were conducted to assess the predictive value of TMB for breast cancer survival.

## Materials and methods

This systematic review and meta-analysis were in accordance with the Preferred Reporting Items for Systematic Reviews and Meta-Analyses (PRISMA) [23]. This protocol was registered with the International Prospective Register of Systematic Reviews (PROSPERO CRD42022342488).

### Literature search

We searched the databases PubMed, Embase, Web of Science, and Cochrane Library for articles published through May 31, 2022. The search terms were (mutational burden OR mutation burden OR mutational load OR mutation load OR TMB OR TML) AND (breast cancer OR breast neoplasms OR breast tumor OR cancer of breast OR human mammary neoplasm OR human mammary carcinomas). Subsequently, to collect as many pertinent studies as possible, we consulted the references of the identified articles. Conference abstracts published in the American Society of Clinical Oncology (ASCO) and the European Society for Medical Oncology (ESMO) were also searched for relevant studies.

### Study selection

The two researchers (KLY and LS) independently searched for and screened the articles based on the inclusion criteria. If their opinions differ, the third researcher will be asked to render a verdict (CHX).

We conducted the research using the following criteria for inclusion: (1) Patients were diagnosed with breast cancer. (2) A clear TMB cut-off value was used to divide patients into two groups: those with high TMB levels and those with low TMB levels. (3) The clinical outcome was overall survival (OS) or progression-free survival (PFS). (4) The studies provided the hazard ratio (HR) and its 95% confidence interval (95% CI) related to the TMB level. In the absence of the aforementioned data, studies were required to provide Kaplan–Meier curves or original data to calculate HR and 95% CI. (5) Each study was composed in English.

The following criteria determined exclusion: TMB consisted of three layers, incomplete data, reviews, meta-analyses, animal studies, fundamental studies, editorials, comments, and case reports.

### Data extraction and quality assessment

Each study's experiment information was collected as follows: title, first author, published year, type of study, region, sample size, breast cancer subtype, type of therapy, sample source, TMB detection method, TMB cut-off value, median TMB value and its range, clinical outcome, HR, and 95% CI.

All studies were cohort studies, and the Newcastle–Ottawa Scale (NOS) was utilized to evaluate their quality. Each study was assessed from three aspects with a total score of 0–9. And the studies were divided into three grades based on their cumulative score, 8–9 as high-quality, 5–7 as intermediate-quality, and 0–4 as low-quality [24].

### Statistical methods and data analysis

To assess the prognostic value of TMB on survival of breast cancer, we compared the different survival benefits between high and low TMB groups using pooled HR. All data analyses were calculated by STATA 16.0. When heterogeneity was significant, we conducted pooled HR utilizing the random-effects model; otherwise, the fixed-effects model was used. Heterogeneity was evaluated by the *I*^*2*^ statistic and *p*-value, with *I*^*2*^ > 50% and *p* < 0.1 as significant heterogeneity [25].

In the meantime, the origin of the heterogeneity was investigated further by means of publication bias assessment, sensitivity analysis, and subgroup analysis. We drew the funnel plot to estimate publication bias and conducted Begg’s test to quantify the funnel plot. The publication bias was absent if the funnel plot was symmetrical and *P* > 0.05 in Begg’s test [26]. A sensitivity analysis was conducted to examine the impact of removing each study from the pooled HR. Type of therapy, breast cancer subtype, TMB detection method, TMB cutoff value, and sample source was considered in subgroup analysis. For the studies of replacing HR with Kaplan–Meier curves, HR and corresponding 95% CI was calculated through program files provided by Tierney et al. using the survival data, extracted from Kaplan–Meier curves by a tool named Engauge Digitizer [27]. In accordance with the GRADE criteria for assigning a grade of evidence, the quality of evidence for main outcomes was determined after contemplating study design, study quality, consistency, and directness [28].

## Results

### Literature search

**Fig. 1:**
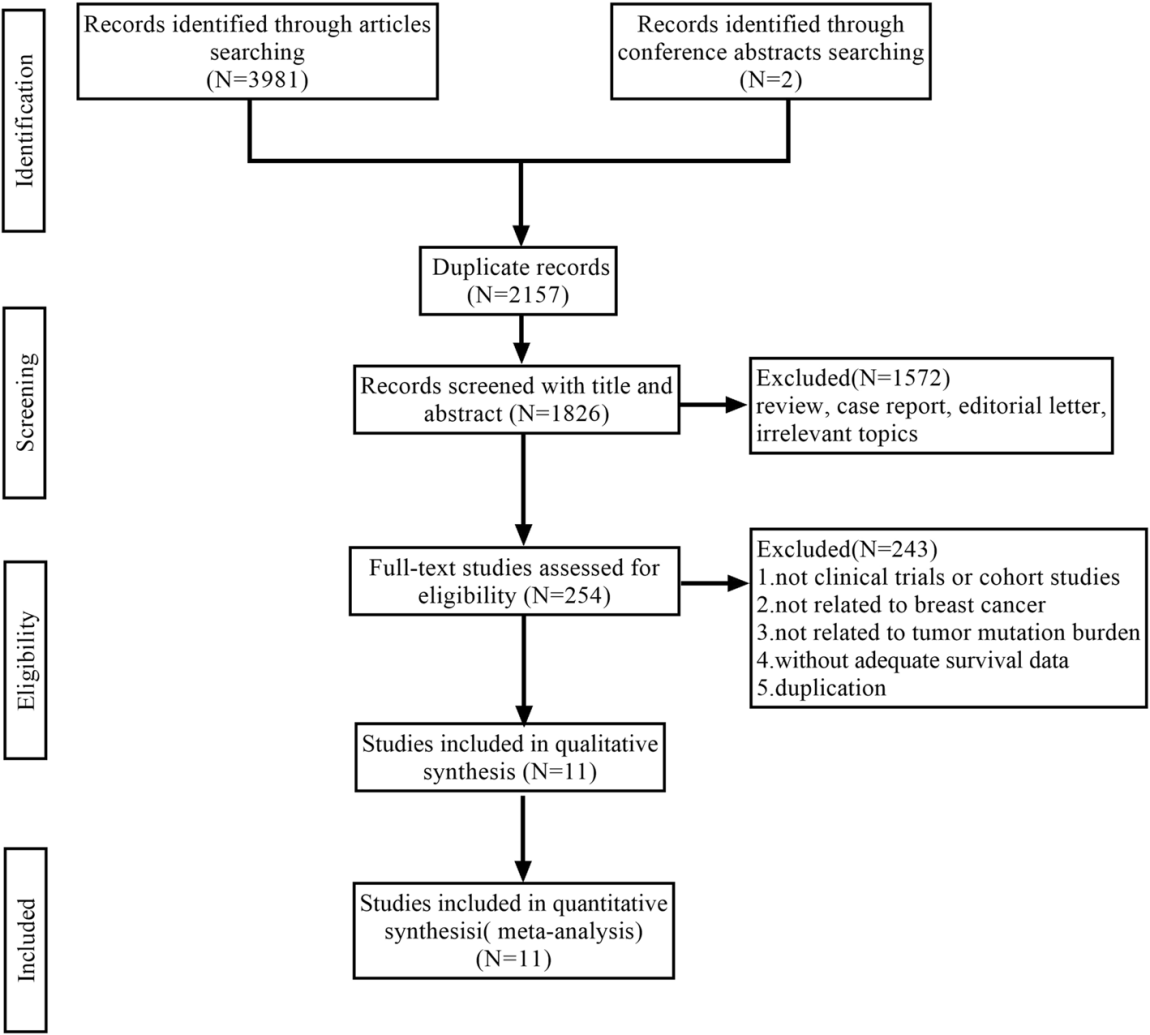
The flow diagram of the study selection process

We collected a total of 3983 potential articles via two investigators largely independent searching. After eliminating duplicates, we used the title and abstract to identify the literature.1572 articles were excluded due to the fact that they classified the review, case report, editorial letter, and irrelevant topics. Subsequently, 254 articles were eligible for preliminary screening. Finally, 11 studies were included among the 254 eligible full-text articles. The flowchart for searching and identifying literature is presented in Fig. 1.

### Study characteristics and quality assessment

The sixteen cohorts from eleven studies were included, with a total of 1,722 patients. The baseline characteristics of patients, type of therapy, and TMB-relevant information was recorded at length in Table 1. All were retrospective cohort studies, with four reported in conference abstracts and twelve in articles. There were three studies (Liao [29], Anwar [30], and Emens [12]) conducted multiple subgroup trials involving different breast cancer subtypes.

**Table 1:** The main characteristics of studies included in the meta-analysis

Author	Year	Type of study	Region	Breast cancer subtype	Number of patients ( high / low TMB)	Type of therapy	Outcome	Sample source	TMB detection method	TMB cutoff value	Median / average* TMB (Range)
Wen [31]	2022	Article	Multiple areas	HER2 +	216 ( 43 / 173)	Chemotherapy, Chemotherapy plus HER2 + targeted therapy	OS	tissue	NA	3.05 Mut/Mb	3.05 Mut/Mb*
Liao(a) [29]	2022	Article	China	breast cancer	OS:139 ( 36 / 103) PFS:128 ( 29 / 99)	Chemotherapy, Endocrine therapy, Radiotherapy	OS, PFS	blood	NGS (PredicineCARE)	4.3 Mut/Mb	24.8 Mut/Mb ( 0–324)
Liao(b) [29]	2022	Article	China	HER2 +	39 ( 13 / 26)	Chemotherapy, Endocrine therapy, Radiotherapy	OS	blood	NGS (PredicineCARE)	4.3 Mut/Mb	NA
Liao(c) [29]	2022	Article	China	TNBC	34 ( 8 / 26)	Chemotherapy, Endocrine therapy, Radiotherapy	OS	blood	NGS (PredicineCARE)	4.3 Mut/Mb	NA
Makhlin [32]	2021	Conference abstratct	Multiple areas	breast cancer	188 ( 13 / 175)	NA	OS	tissue	NA	3 mutations	2.2mutations* ( NA)
Gao [33]	2021	Article	Multiple areas	TNBC	101 ( 49 / 52)	NA	OS	tissue	NA	1.26 Mut/Mb	1.26 Mut/Mb ( 0.05–28.03)
Anwar(a) [30]	2021	Article	China	HER2 +	28 ( 8 / 20)	HER2 + targeted therapy	OS, PFS	blood	NGS(OncoMD/OncoMD-Plus)	5.0 Mut/Mb	3.0 Mut/Mb (0–22)
Anwar(b) [30]	2021	Article	China	HER2 + with brain metastasis	8 ( 6 / 2)	HER2 + targeted therapy	OS, PFS	blood	NGS(OncoMD/OncoMD-Plus)	5.0 Mut/Mb	NA
Chen [21]	2020	Article	China	HER2 +	28 ( 8 / 20)	HER2 + targeted therapy	PFS	blood	NGS(OncoMD/OncoMD-Plus)	5.0 Mut/Mb	3.0 Mut/Mb (0–22)
Emens(a) [12]	2020	Conference abstratct	Multiple areas	TNBC	579 ( 144 / 435)	ICIs	OS, PFS	tissue	NGS (FoundationOne)	7.02 Mut/Mb	4.39 Mut/Mb(0–46.51)
Emens(b) [12]	2020	Conference abstratct	Multiple areas	TNBC (PD-L1-)	NA	ICIs	PFS	tissue	NGS (FoundationOne)	7.02 Mut/Mb	NA
Emens(c) [12]	2020	Conference abstratct	Multiple areas	TNBC (PD-L1 +)	NA	ICIs	PFS	tissue	NGS (FoundationOne)	7.02 Mut/Mb	NA
Barroso-Sousa [17]	2020	Article	Multiple areas	TNBC	62 ( 12 / 50)	ICIs, ICIs plus Chemotherapy/ Targeted therapy	OS, PFS	tissue	NGS (OncoPanel)	10 Mut/Mb	6 Mut/Mb ( NA)
Li [34]	2020	Article	Multiple areas	breast cancer	44 (10% / 90%)	ICIs	OS	tissue /blood	NGS (MSK-IMPACT)	6.8 Muts/MB	NA
Samstein [22]	2020	Article	Multiple areas	breast cancer	45 ( 12 / 33)	ICIs	OS	blood	NGS (MSK-IMPACT)	5.9 Muts/MB	3.94 Mut/Mb (0–46.52)
Park [13]	2018	Article	Korea	HER2 +	OS: 46 ( 16 / 30) PFS: 37 ( 13 / 24)	Chemotherapy plus HER2 + Targeted therapy	OS, PFS	tissue	WES	100 mutations	NA

*represented the value was average value

NOS only evaluated 12 cohorts for bias risk evaluation, because 4 cohorts from conference abstracts were not applicable. The results indicated 4 trials were of high quality and 8 trials were medium. The inclusion of the four cohorts described in the conference abstracts may diminish the overall quality of the included studies. And the four cohorts were comprised of hundreds of patients who provided independent cases and controls, ascertainment of exposure, and complete experimental results. We presume that it is essential to include these four cohorts in the meta-analysis, as they contributed significantly to the pooled result.

### Pooled effects of HR for OS and PFS

**Fig. 2:**
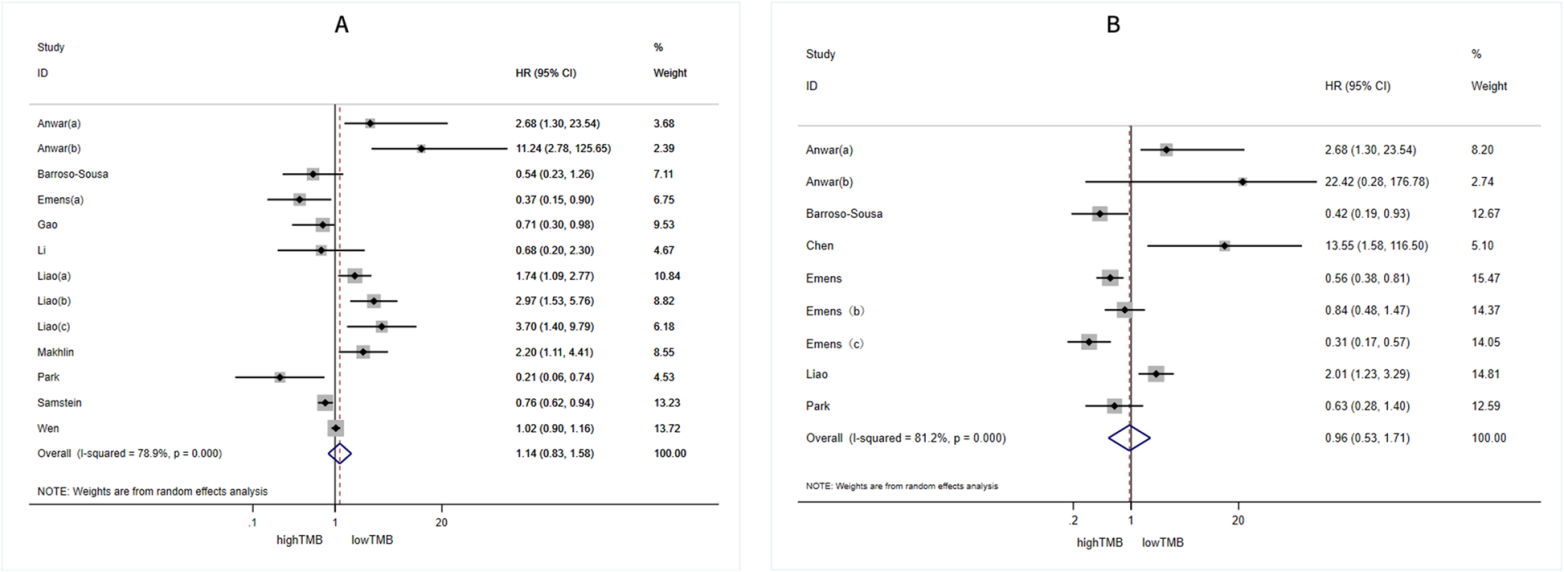
Forest plots of HR for OS (**A**) and HR for PFS (**B**) in patients with high TMB versus low TMB

We calculated the pooled effects of HR for OS extracted from 13 cohorts, and for PFS extracted from 9 cohorts. The pooled effects of HR for both OS ( HR: 1.14, 95% CI: 0.83–1.58; *p* > 0.01) and PFS ( HR: 0.96, 95% CI: 0.53–1.71; *p* > 0.01) indicated there is no significant difference in survival benefits between groups with high and low TMB (Figs. 2). Moreover, the results further demonstrated high heterogeneity among the combined studies (*I*^*2*^ = 78.9% and 81.2%, respectively). To ascertain forward the origin of heterogeneity among the included studies, a subgroup analysis was conducted.

### Subgroup analysis for OS

The results of the subgroup analysis for OS were depicted in Table 2 and Supplementary Fig. 1, 2, 3, 4 and 5. In the ICIs subgroup, the high TMB group displayed more benefits of OS (HR = 0.72, 95% CI: 0.59,0.87, *p* = 0.001), although the variation was not statistical in the non-ICIs subgroup (HR = 1.76, 95% CI: 0.97,3.20, *p* = 0.062). For sample source subgroup analysis, when the sample source was blood, the OS of patients with high TMB was shorter than patients with low TMB (HR = 2.25, 95% CI: 1.09,4.64, *p* = 0.028). In terms of the tumor tissue subgroup, there was no statistically significant difference between the high and low TMB patient groups. (HR = 0.74, 95% CI: 0.45,1.21, *p* = 0.227). We conducted a subgroup analysis according to the median TMB cutoff value. In the TMB cutoff value > 5 Mut/Mb subgroup, more OS benefits were observed in patients with high TMB levels (HR: 0.72, 95% CI: 0.59,0.87, *p* = 0.001), while a worse OS was found in TMB cutoff value ≤ 5 Mut/Mb subgroup (HR = 1.81, 95% CI: 1.10,2.98, *p* = 0.02). Meanwhile, the heterogeneity of the ICIs subgroup and TMB cutoff value > 5 Mut/Mb subgroup were drastically reduced by subgroup analysis. The benefit of OS related to TMB level had little correlation with the pathological classification of breast cancer and TMB detection method.

**Table 2:** The subgroup analysis in OS of patients with high TMB compared to low TMB

Subgroup	Number of studies	Effects model	Pooled ES		Heterogeneity	
			**HR [95% CI]**	***P***	***I***^***2***^	***P*-Value**
Type of therapy						
ICIs	4	Fixed	0.72[0.59,0.87]	0.001	0%	0.419
Non-ICIs	7	Random	1.76[0.97,3.20]	0.062	82%	< 0.001
Breast cancer subtype						
HER2 +	5	Random	1.57[0.65,3.82]	0.319	82.9%	< 0.001
TNBC	4	Random	0.83[0.35,1.95]	0.668	77.5%	0.004
Sample source						
tumor	6	Random	0.74[0.45,1.21]	0.227	73.7%	0.002
blood	6	Random	2.25[1.09,4.64]	0.028	86.6%	< 0.001
TMB detection method						
NGS	9	Random	1.37[0.78,2.39]	0.271	82%	< 0.001
WES	1	-	0.21[0.06,0.74]	-	-	-
TMB cutoff value						
≤ 5 Mut/Mb	7	Random	1.81[1.10,2.98]	0.02	79.2%	< 0.001
> 5 Mut/Mb	4	Fixed	0.72[0.59,0.87]	0.001	0%	0.419

### Subgroup analysis for PFS

The results of the subgroup analysis for PFS were exhibited in Table 3 and Supplementary Figs. 6, 7, 8, 9 and 10. The type of therapy subgroup analysis indicated that patients treated with ICIs therapy in the high TMB group similarly showed better PFS (HR = 0.52, 95% CI: 0.35,0.77, *p* < 0.001). And in the TNBC subgroup, more benefits of PFS were obtained in the high TMB group (HR: 0.52, 95% CI: 0.35,0.77, *p* = 0.001). The subgroup analysis based on sample source revealed a correlation between elevated TMB and a longer PFS when the sample source was tumor tissue (HR: 0.53, 95% CI: 0.38,0.74, *p* < 0.001), while shorter PFS when it was blood (HR: 3.37, 95% CI: 1.34,8.44, *p* = 0.01). In the TMB cutoff value > 5 Mut/Mb subgroup, longer PFS was observed in high TMB group (HR: 0.52, 95% CI: 0.35,0.77, *p* = 0.001), while shorter PFS was obtained in high TMB group on the condition that the TMB cutoff value ≤ 5 Mut/Mb (HR: 3.37, 95% CI: 1.34,8.44, *p* = 0.01). Through subgroup analysis, the heterogeneity of each subgroup was significantly reduced. The correlation between TMB level and PFS was unrelated to the TMB detection method.

**Table 3:** The subgroup analysis in PFS of patients with high TMB compared to low TMB

Subgroup	Number of study	Effects model	Pooled ES		Heterogeneity	
			**HR [95% CI]**	***P***	***I***^***2***^	***P*-Value**
Type of therapy						
ICIs	4	Fixed	0.52[0.35,0.77]	< 0.001	50.3%	0.110
Non-ICIs	5	Random	2.31[0.89,5.97]	0.086	68.5%	0.013
Breast cancer subtype						
HER2 +	4	Random	3.26[0.64,16.71]	0.156	74.2%	0.009
TNBC	4	Fixed	0.52[0.35,0.77]	0.001	50.3%	0.110
Sample source						
tumor	5	Fixed	0.53[0.38,0.74]	< 0.001	35.4%	0.185
blood	4	Fixed	3.37[1.34,8.44]	0.01	38.1%	0.183
TMB detection method						
NGS	8	Random	1.04[0.54,2.01]	0.909	83.4%	< 0.001
WES	1	-	0.63[0.28,1.14]	-	-	-
TMB cutoff value						
≤ 5 Mut/Mb	4	Fixed	3.37[1.34,8.44]	0.01	38.1%	0.183
> 5 Mut/Mb	4	Fixed	0.52[0.35,0.77]	0.001	50.3%	0.110

### Sensitivity analysis and publication bias

**Fig. 3:**
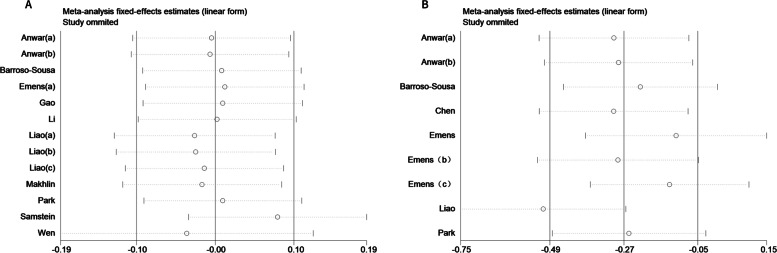
Sensitivity analysis of pooled effects for OS (**A**) and PFS (**B**)

**Fig. 4:**
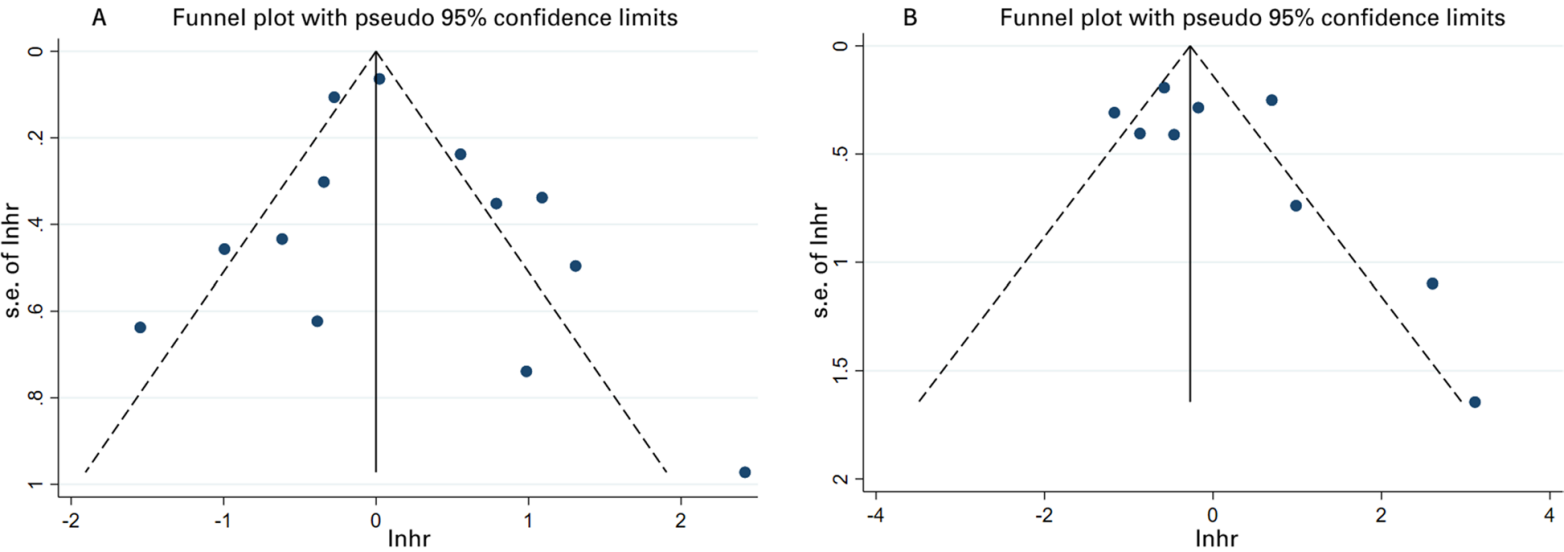
The funnel plots for OS (**A**) and PFS (**B**)

Sensitivity analysis exhibited little variation in the pooled effects after excluding each article in turn (Fig. 3). The symmetric funnel plots (Fig. 4) and Begg’s test (OS: *P* = 0.855, PFS: *P* = 0.251) suggested publication bias was absent. In accordance with the GRADE criteria, the quality of evidence for OS and PFS was low.

## Discussion

Our study summarized the effects of HR collected from 1,722 breast cancer patients who were treated with immunotherapy, chemotherapy, targeted therapy, etc. The findings indicated that compared with the low TMB group, the advantage of OS and PFS in the high TMB group was not obvious. Moreover, both the OS and PFS analyses revealed a high degree of heterogeneity among the included studies. The results of subgroup analysis for OS exhibited that different types of therapy and TMB cutoff values may be the explanations for high heterogeneity. In subgroup analysis for PFS, the heterogeneity decreased substantially in other subgroups except for the TMB detection method subgroup.

Although the association between TMB and the survival of patients with melanoma, lung, and colon cancer has been studied, the relationship in breast cancer needs to be verified [35–37]. Our meta-analysis has contributed to supplementing to the prognostic role of TMB on survival in breast cancer. Thomas et al. demonstrated that TMB was a factor in breast cancer patients' immune-mediated survival [38]. The favorable immune-infiltrate dispositions subclass was associated with prolonged survival of patients with high TMB. Our subgroup analysis exhibited that for the ICIs treatment subgroup the benefit of survival increased in the high TMB group, whereas this trend was not applied to non-ICIs treatment. No matter whether patients who treated with chemotherapy or HER-2 targeted therapy, higher TMB was significantly correlated with worse OS or PFS. Park’s team proposed a hypothesis that TMB produced new neoantigens to increase T cell target, thus patients with high TMB could also benefit from HER2 target therapy and chemotherapy [13]. Moreover, studies have reported that HER2-targeted agents, such as trastuzumab, can affect response by regulating immune activity [39]. In our subgroup analysis, we selected ICIs and ICIs in combination with chemotherapy or targeted therapy. Therefore, we support TMB as a biomarker to screen more potential people who may benefit from ICIs, and ICIs combined with chemotherapy or targeted therapy as a new research direction in breast cancer.

To our knowledge, there are still many controversies about the prediction of TMB on the survival of HER2 + breast cancer. A recent bioinformatics study revealed that the survival rate of HER2 + metastatic breast cancer was lower in the group with low TMB than in the group with high TMB [13]. In another HER2 + breast cancer article, Wen reported that higher TMB was associated with shorter OS [31]. Our study suggested patients in the high TMB group appeared to have a shorter survival rate for HER2 + breast cancer, despite the fact that there was no statistically significant difference between the two groups. The early study reported that TNBC exhibited more immunogenic characteristics than HER2 + breast cancer [40]. On the basis of the IMpassion130 study and exploratory analysis, improved PFS related to high TMB in TNBC no matter the PD-L1 expression level [41]. Moreover, the Gao team showed that the clinical outcome of TNBC in the high TMB group is better even without immunotherapy [33]. In our subgroup analysis, PFS in the high TMB group is prolonged with a statistically significant difference in TNBC. To provide more treatment options for TNBC, the prognostic value of TMB on survival of TNBC is still worth exploring in further study.

Although the landscape of TMB was revealed in some studies for reference, the issue that how to establish the cutoff value of TMB in breast cancer is still critical [42]. As early as 2020, FDA approved pembrolizumab for advanced solid tumors as the cutoff value of TMB was ≥ 10 Mut/Mb [15]. Despite this, we cannot define a universal cutoff value to predict the survival of diverse tumors, as the difference in TMB value between different tumors is very large, even up to 1,000 times [22, 43]. There are many methods to define the cutoff value of TMB, such as median value, 75th percentile value, and FDA approval standard 10 Mut/Mb. In accordance with the landscape of TMB composed of 100,000 human cancer genomes, Goodman et al. defined low TMB as ≤ 5 Mut/Mb [44]. The TMB thresholds reported in the included studies ranged from 1.26 Mut/Mb to 10 Mut/Mb, with a median value of 5 Mut/Mb. We divided the studies into two subgroups by the threshold of 5 Mut/Mb. The result revealed that the PFS of patients with high TMB was considerably longer than low TMB in the TMB cutoff value > 5Mut/Mb subgroup, and the result was the opposite in the TMB cutoff value ≤ 5 Mut/Mb subgroup. And yet, the heterogeneity has declined substantially in this subgroup analysis. The value 5 Mut/Mb may provide a reference for the choice of TMB cutoff value in future breast cancer trials.

The sample source of TMB detection was usually tumor tissue or blood. Tissue TMB (tTMB) is employed commonly as most patients could get tissues for gene sequencing. Furthermore, blood TMB (bTMB) is an attractive alternative if tTMB is not able to be evaluated for lack of sufficient quantities. bTMB is the number of mutations of ctDNA (circulating tumor deoxyribonucleic acid) produced during tumor cell degradation [45]. The quantity and quality of ctDNA will directly influence the value of bTMB; therefore, ctDNA is a crucial factor in determining the accuracy of a test. When ctDNA is derived from hemopoiesis or nonneoplastic lesions, the consequence is highly heterogeneous [46]. The number of mutations calculated by tTMB included insertion mutations, deletion mutations, and single nucleotide variant mutations, while bTMB included only single nucleotide variant mutations [47]. This is yet another justification for the heterogeneity between tTMB and bTMB. A series of previous studies have shown that tTMB and bTMB are positively correlated [48–50]. However, our study revealed that the prognostic role of tTMB and bTMB on survival was the opposite. For the relation between bTMB and tTMB, an in-depth research is still required.

The detection method of TMB includes whole exome sequencing (WES) and next-generation sequencing (NGS), with upsides and downsides for both. Since the golden standard of TMB detection has been on the basis of the WES, much research about TMB is carried out by using WES technology. Nonetheless, the application of WES is restricted to a certain extent in clinical because it is expensive and complicated to operate [51]. In comparison with WES, NGS has lower cost and more convenient operation, therefore it is more suitable for clinical use. Moreover, the detection results of NGS and WES are correlated, which is another reason for substitutability [52]. An NGS panel containing hundreds of genes is rapidly sequenced with ultrahigh throughput [53, 54]. Although subgroup analysis of the detection method was conducted in our study, we cannot compare the difference between these two sequencing methods due to NGS being adopted in most studies except for one study. Substantial heterogeneity exists between various panels within the NGS subgroup, indicating that different targeted NGS panels should also be standardized.

This study is the first meta-analysis that investigated the relationship of TMB with the survival of breast cancer, which provides a reference for TMB studies in the future. In this meta-analysis, we calculated pooled HR to assess the prognostic value of TMB on breast cancer survival. However, several limitations of our analysis should be considered. Initially, a few important clinical characteristics, namely, age, menopause, surgery history, and combination therapy were not available to analyze in our study, which may affect the heterogeneity. Secondly, in the included studies, TMB was computed by a variety of methods. The majority of the studies reported the TMB calculation method in a unit of Mut/Mb and two studies in a unit of mutations. Thirdly, we excluded studies that separated TMB into three layers (high, medium, and low), resulting in publication bias. Despite the limitations, we observed most sources of heterogeneity through subgroup analysis.

## Conclusions

Our meta-analysis indicated that TMB as a prognostic biomarker is not generally applicable in breast cancer. The high TMB may be associated with prolonged survival only in ICIs therapy, nonetheless, the relation is not obvious in non-ICIs therapy. We endorse the use of TMB as a prognostic biomarker to identify more patients who may benefit from ICIs, ICIs in combination with chemotherapy, or targeted therapy. In addition, TNBC patients with high TMB tend to gain more clinical benefits than other breast cancer subtypes. We recommend that in TMB detection we may give preference to tumor tissue and cut-off value > 5Mut/Mb.

## Supplementary Information

**Additional file 1: Supplementary Figure 1.** Forest plot of subgroup analysis for OS on type of therapy.

**Additional file 2: Supplementary Figure 2.** Forest plot of subgroup analysis for OS on breast cancer subtype.

**Additional file 3: Supplementary Figure 3.** Forest plot of subgroup analysis for OS on sample source.

**Additional file 4: Supplementary Figure 4.** Forest plot of subgroup analysis for OS on TMB detection method.

**Additional file 5: Supplementary Figure 5.** Forest plot of subgroup analysis for OS on TMB cutoff value.

**Additional file 6: Supplementary Figure 6.** Forest plot of subgroup analysis for PFS on type of therapy.

**Additional file 7: Supplementary Figure 7.** Forest plot of subgroup analysis for PFS on breast cancer subtype.

**Additional file 8: Supplementary Figure 8.** Forest plot of subgroup analysis for PFS on sample source.

**Additional file 9: Supplementary Figure 9.** Forest plot of subgroup analysis for PFS on on TMB detection method.

**Additional file 10: Supplementary Figure 10.** Forest plot of subgroup analysis for PFS on TMB cutoff value.

## Data Availability

All datasets generated for this study are included in the article/Supplementary Material.
