# Supplementary figures and images for: The prognostic role of tumor mutation burden on survival of breast cancer: a systematic review and meta-analysis
Source: BMC Cancer. 2022 Nov 17;22:1185. doi: 10.1186/s12885-022-10284-1 (PMC9673350; doi:10.1186/s12885-022-10284-1)

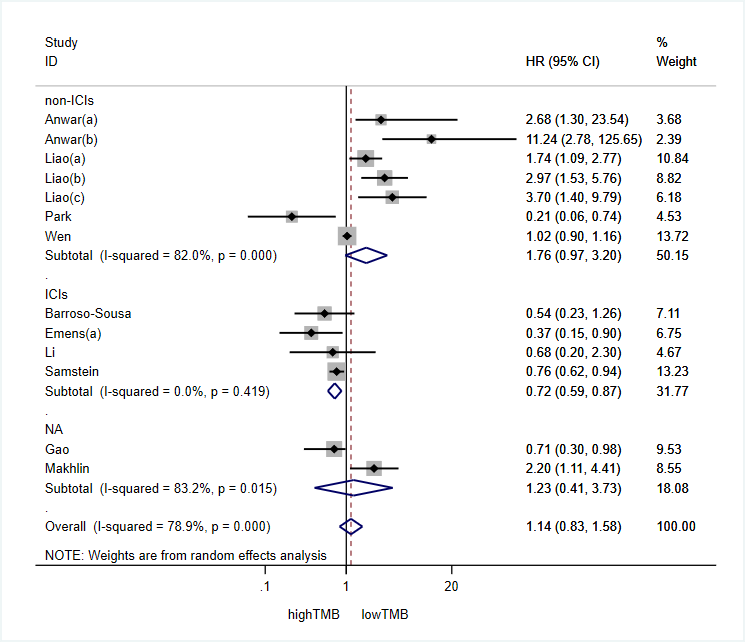

Supplement: Supplementary file 1 — Additional file 1: Supplementary Figure 1. Forest plot of subgroup analysis for OS on type of therapy. [file 12885_2022_10284_MOESM1_ESM.tif]

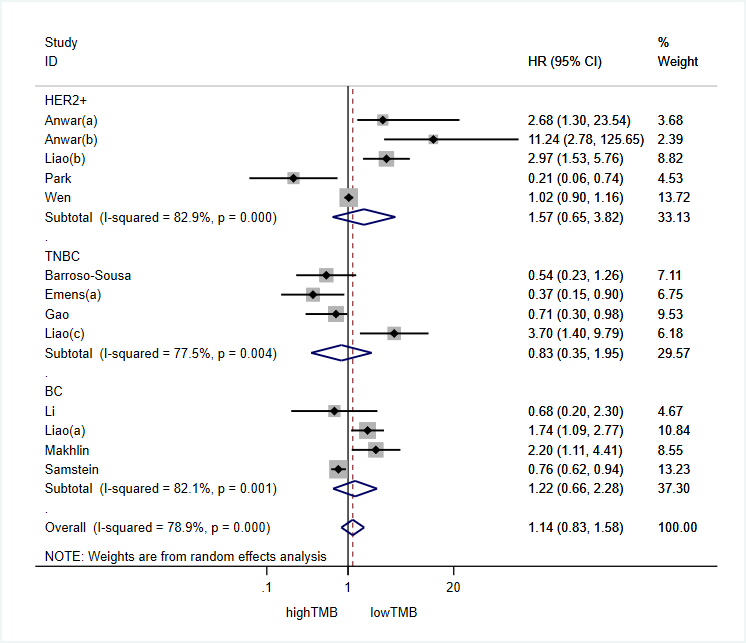

Supplement: Supplementary file 2 — Additional file 2: Supplementary Figure 2. Forest plot of subgroup analysis for OS on breast cancer subtype. [file 12885_2022_10284_MOESM2_ESM.tif]

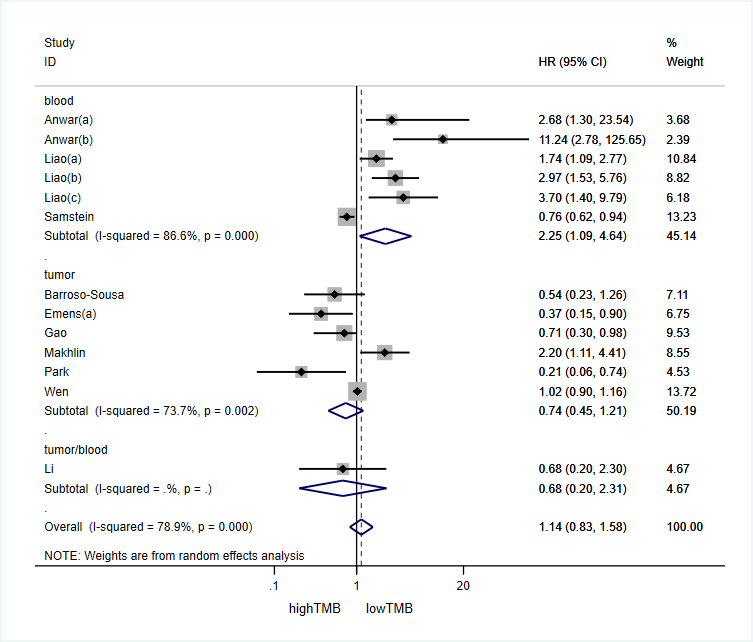

Supplement: Supplementary file 3 — Additional file 3: Supplementary Figure 3. Forest plot of subgroup analysis for OS on sample source. [file 12885_2022_10284_MOESM3_ESM.tif]

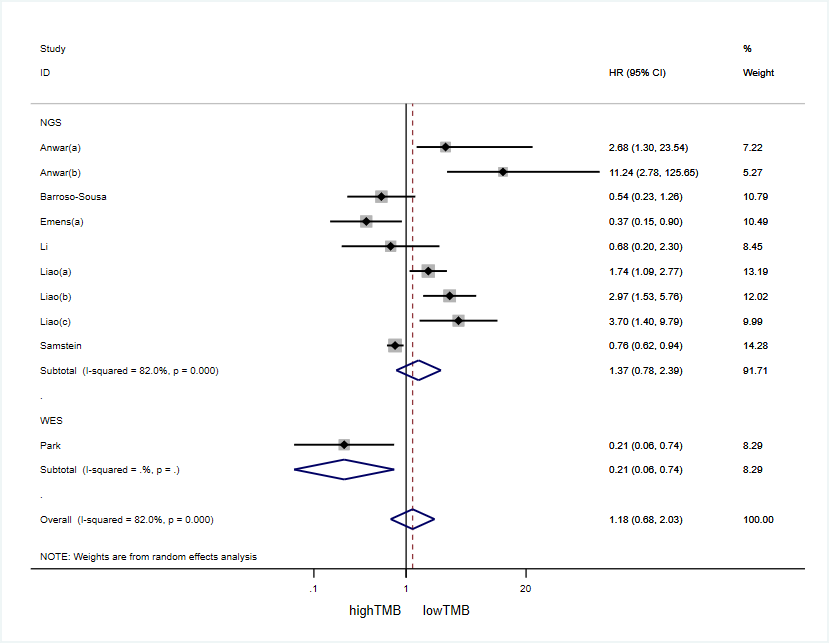

Supplement: Supplementary file 4 — Additional file 4: Supplementary Figure 4. Forest plot of subgroup analysis for OS on TMB detection method. [file 12885_2022_10284_MOESM4_ESM.tif]

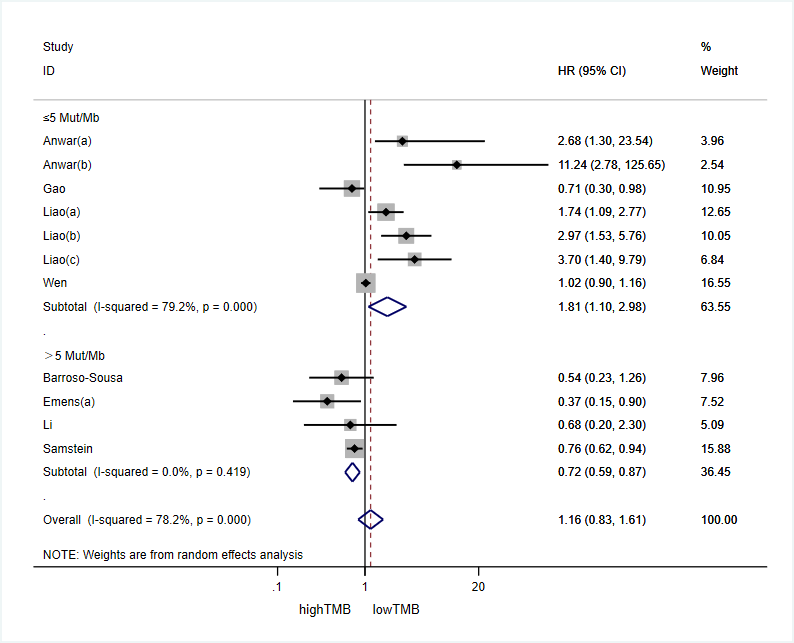

Supplement: Supplementary file 5 — Additional file 5: Supplementary Figure 5. Forest plot of subgroup analysis for OS on TMB cutoff value. [file 12885_2022_10284_MOESM5_ESM.tif]

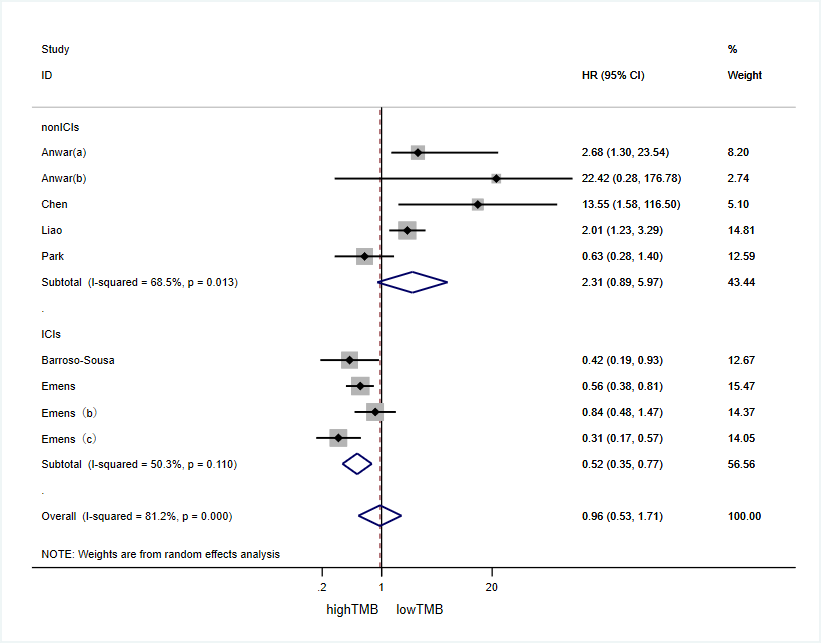

Supplement: Supplementary file 6 — Additional file 6: Supplementary Figure 6. Forest plot of subgroup analysis for PFS on type of therapy. [file 12885_2022_10284_MOESM6_ESM.tif]

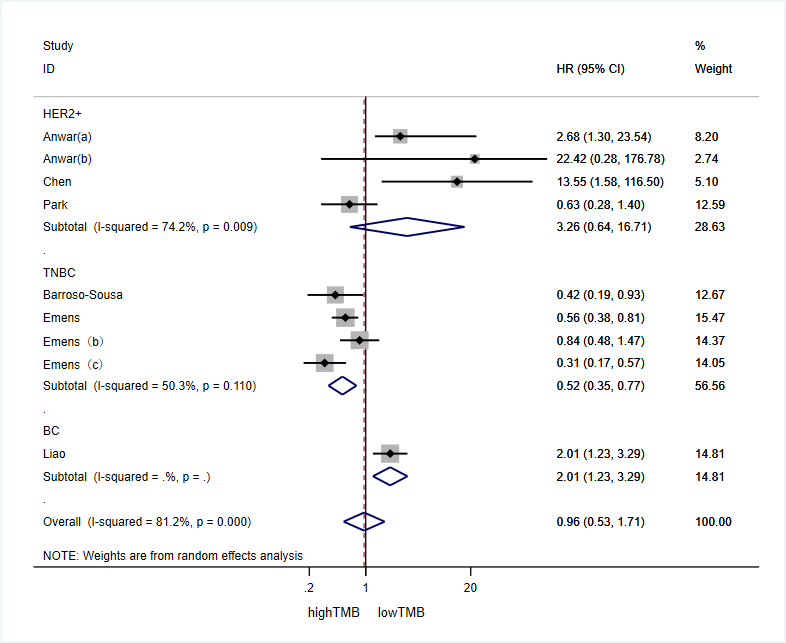

Supplement: Supplementary file 7 — Additional file 7: Supplementary Figure 7. Forest plot of subgroup analysis for PFS on breast cancer subtype. [file 12885_2022_10284_MOESM7_ESM.tif]

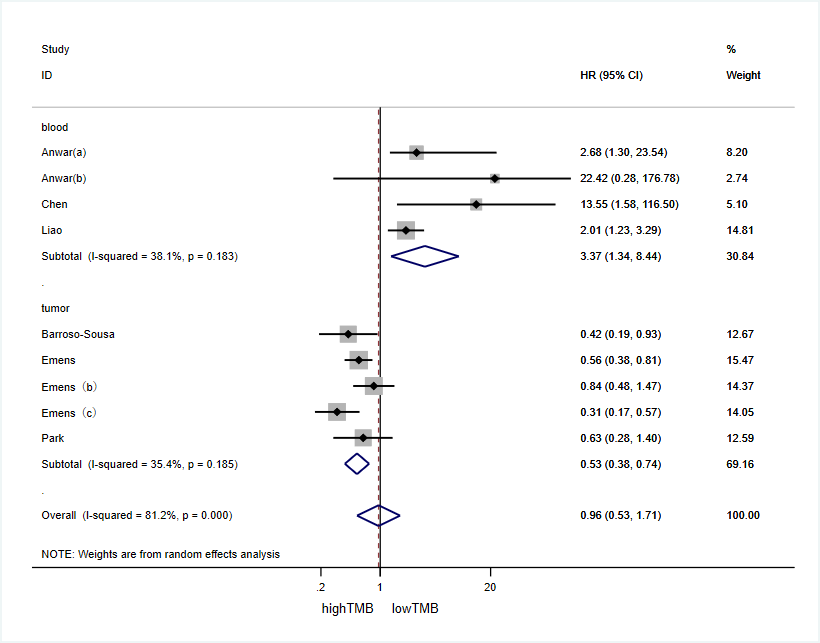

Supplement: Supplementary file 8 — Additional file 8: Supplementary Figure 8. Forest plot of subgroup analysis for PFS on sample source. [file 12885_2022_10284_MOESM8_ESM.tif]

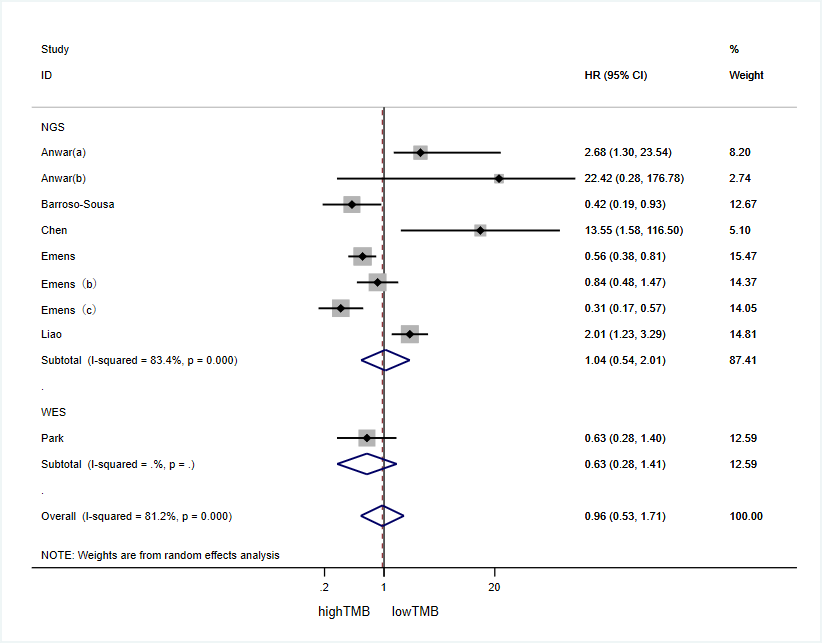

Supplement: Supplementary file 9 — Additional file 9: Supplementary Figure 9. Forest plot of subgroup analysis for PFS on on TMB detection method. [file 12885_2022_10284_MOESM9_ESM.tif]

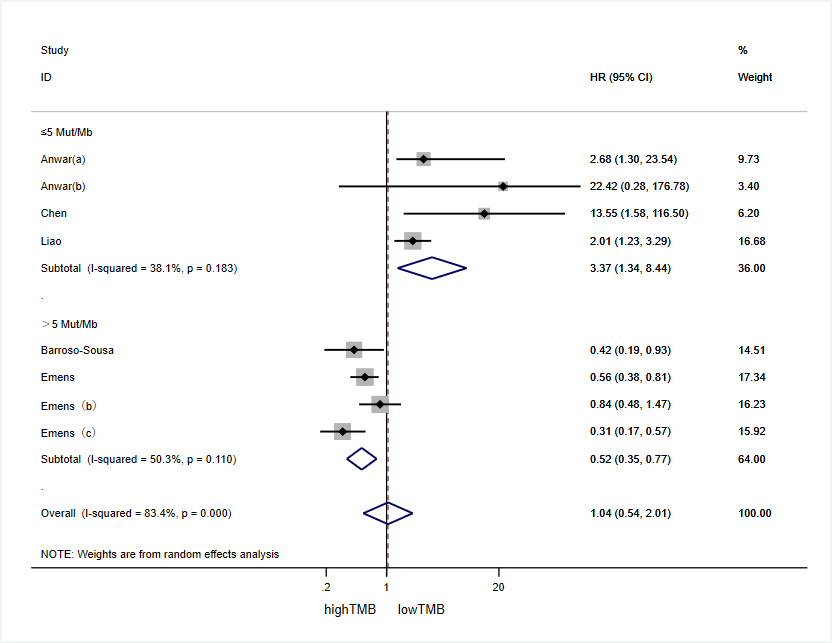

Supplement: Supplementary file 10 — Additional file 10: Supplementary Figure 10. Forest plot of subgroup analysis for PFS on TMB cutoff value. [file 12885_2022_10284_MOESM10_ESM.tif]
